# Use of large language models to identify pseudo‐information: Implications for health information

**DOI:** 10.1111/hir.12569

**Published:** 2025-03-20

**Authors:** Boris Schmitz

**Affiliations:** ^1^ Department of Rehabilitation Sciences Faculty of Health, University of Witten/Herdecke Witten Germany; ^2^ DRV Clinic Königsfeld Center for Medical Rehabilitation Ennepetal Germany

**Keywords:** Artificial Intelligence (AI), consumer health information, internet, misinformation, patient information

## Abstract

**Background:**

Open‐access scientific research is an essential source of health‐related information and self‐education. Artificial intelligence‐based large language models (LMMs) may be used to identify erroneous health information.

**Objective:**

To investigate to what extent LMMs can be used to identify pseudo‐information.

**Methods:**

Four common LMM applications (ChatGPT‐4o, Claude 3.5 Sonnet, Gemini and Copilot) were used to investigate their capability to indicate erroneous information provided in an open‐access article.

**Results:**

Initially, ChatGPT‐4o and Claude were able to mark the provided article as an unreliable information source, identifying most of the inaccuracy problems. The assessments provided by Gemini and Copilot were inaccurate, as several critical aspects were not identified or were misinterpreted. During the validation phase, the initially accurate assessment of ChatGPT‐4o was not reproducible, and only Claude was able to detect several critical issues in this phase. The verdicts of Copilot and Gemini remained largely unaltered.

**Discussion:**

Large heterogeneity exists between LMMs in identifying inaccurate pseudo‐information. Replication in LMM output may constitute a significant hurdle in their application.

**Conclusion:**

The accuracy of LMMs needs to be further improved until they can be reliably used by patients for health‐related online information and as assistant tools for health information and library services workers without restriction.

**Key Messages**

- Online resources including open‐access science are an important source of health information; however, erroneous or pseudo‐information can be harmful
- AI‐based LMMs have been suggested to improve access for the general public to health‐related content.
- In general, LMMs appear capable of identifying pseudo‐information accurately, but large heterogeneity between LMMs exists, and reproducibility is low.
- Today, LMMs cannot replace critical thinking when it comes to the identification of pseudo‐information.
- Training programs on AI‐based LMMs should be offered to users in health knowledge services.

## INTRODUCTION

In the digital age, with health information being readily accessible and rapidly disseminated, ensuring scientific accuracy and rigour is of central importance. The general public relies on online sources for health‐related information, making it crucial that the information provided is accurate, reliable, and evidence‐based. Misinformation, especially during a global health crisis such as the COVID‐19 pandemic, can lead to harmful consequences, including poor health outcomes and increased anxiety (Choukou et al., 2022). While it is the responsibility of scientists, healthcare professionals and digital content providers including scientific journals to uphold the highest standards of accuracy and rigour in their communications, the health literacy of readers (Berkman et al., 2010) is the final checkpoint for identifying misinformation or even fraudulent content (disinformation). Artificial intelligence (AI) is a rapidly advancing tool not only in multiple research areas including healthcare but has also been suggested to assist communication with patients (Moons & Van Bulck, 2024; Zhu et al., 2023). Large AI‐based language models (LMMs) have the potential to help readers assess and understand scientific content, as well as reduce misinterpretation or bias. Health knowledge services and associated specialists may use LMMs as assistant tools in finding, appraising, and summarizing health research also to enable evidence‐based decision‐making by healthcare professionals. In addition, LMMs represent a way to generate summaries adapted to the individual's knowledge level, enabling the reader to access advanced scientific content (Schmitz, 2023). LMMs may thus also be used to identify erroneous health information and pseudo‐science. However, this has so far not been assessed in necessary detail.

Starting in 2016, Tom Spears, a reporter at the Ottawa Citizen, has smuggled in several pseudo‐scientific articles to open‐access journals under the name of Sam Yosemite. On Retraction Watch he stated that ‘… this journal sent out spam asking for volunteers to serve on its editorial board, and I decided to answer under Sam's name just to amuse anyone who happens to look up the ed board. The catch of course is that no one would have a reason to look up the editorial board of such a rancid little publication in the first place. Anyway, once Sam was on the board, they asked him to write an editorial (McCook, 2018). Apparently, Spears had received an editorial board invitation from the Intellectual Consortium of Drug Discovery and Technology Development, Inc. (ICDTD). Spears accepted and was asked to write an editorial. However, the journal made his contribution disappear after realizing that it was a joke. Following up on Spears' idea to point out the ridiculous behaviour of pseudo‐scientific journals, it appears that not all publishers have become aware of his fraudulency. As of today, Sam Yosemite is still editorial board member of the ‘International Journal of Surgery and Invasive Procedures’ and ‘Significances of Bioengineering & Biosciences’ (both at Crimson Publishers) with his made‐up affiliation ‘Institute for Research on Variant‐Reproactive Interstercis, USA’, claiming that his research is supported by the ‘Foghorn Leghorn Foundation’. His fantastic contributions ‘Re‐Examining the Genetic Bottleneck: Atavistic Regression in Acquired Traits Affects the Outcome for Many Subspecies at the Allelic Level’ (Yosemite, 2019) in the Cohesive Journal of Microbiology & Infectious Disease as well as ‘The Next Nobel Prize Must Go to the Eminent Tom Spears’ (Yosemite, 2018) are available to the interested reader. Spears apparently managed to place pseudo‐scientific manuscripts in journals that at least some of the readers may perceive as reliable sources. While being quite entertaining, this story serves as an example to investigate if modern technology involving LMMs could have identified pseudo‐science.

## METHODS

### Phase 1 – Initial analysis

On 24 August 2024, the article ‘Re‐Examining the Genetic Bottleneck: Atavistic Regression in Acquired Traits Affects the Outcome for Many Subspecies at the Allelic Level’ (Yosemite, 2019) was analysed using readily available tools. This specific article was chosen since the author's intention and approach has been documented. The article combines several aspects that mark it as pseudo‐scientific (missing data, statistics, references, made‐up terms and affiliation) while also holding obvious signs of a parody (author name, cartoon references). The text was uploaded to ChatGPT‐4o (OpenAI Inc., USA) and Claude 3.5 Sonnet (free version; Anthropic PBC, USA) followed by the initial prompt ‘perform a critical scan of the article’. The text of the article was also submitted (pasted) to Copilot (free version, setting ‘balanced’; Microsoft, USA) under Microsoft Bing and Google Gemini (free version; Google LLC, USA) using the identical prompt.

### Phase 2 – Prompt permutation and validation

To increase the test size and add semantic variety among the prompts as suggested (Hagendorff, 2024). Copilot was asked to produce 20 permutations of the original prompt with each prompt slightly modifying the expected thoroughness, depth, or aspect of scrutiny in the task (Table S1). ChatGPT‐4o was used for a semantic analysis of the generated prompts. With an interval of 10 days, on 3 September 2024, these prompts were then used for re‐analysis of the article with all four tools (ChatGPT‐4o, Claude 3.5 Sonnet, Copilot, Gemini) using the task description ‘I will upload an article. After I did this, I want you to run a sequence of 20 different prompts on the article. You may not stop until the 20 prompts have been executed. All 20 prompts are equally important. You will then produce a summary of the prompt completion and identify any major differences between them. At last, a final conclusion should be provided’.

## RESULTS

### General

All applications produced summaries of high linguistic quality (Table 1) within <10 s. No syntactical errors and semantic errors were noted. The user‐friendliness of ChatGPT‐4o and Claude was high, since articles could be analysed directly after the upload. All assessments appeared clear and concise with a length between ~320 and 700 words. Claude and Copilot asked if more detailed feedback on some aspects was required. Copilot and Gemini provided suggestions for improvement of the article (not reported here). In phase 1, all LMMs failed to indicate that no citation but one was given in the text [‘Peter et al.’] and that this reference was missing from the article (which has no reference list at all).

**TABLE 1:** Results of the text analysis by four different AI‐based language models.

*ChatGPT*
The article presents itself as a scholarly piece discussing complex genetic phenomena. However, upon closer examination, several critical issues are evident: Jargon and Language Issues: The article is rife with obscure jargon and phrases that do not seem to align with established scientific terminology. Terms like ‘phish’, ‘benthic standard’, ‘dilatory sample sizes’ and ‘retexting’ are either misused or invented, making the text difficult to understand and raising questions about the author's familiarity with the subject matter. Conceptual Clarity: The article's core concepts, such as genetic bottlenecks, atavistic regression, and allele frequency effects, are legitimate areas of study. However, the explanations provided are confusing and lack coherence. The supposed findings, like the impact of minor gene distance changes on study replication power, are presented in a manner that is not scientifically rigorous or clear. Logical Flow: The paper lacks a logical structure that would be expected in a scientific article. The introduction does not clearly set up the problem or hypothesis, and the results section seems disjointed, jumping between different ideas without clear connections. Authenticity and Credibility: The author is listed as ‘Yosemite Sam’, which is a well‐known cartoon character, casting doubt on the authenticity of the article. This, combined with the unusual language and unclear concepts, suggests that the paper may be a parody, hoax, or an attempt at satire rather than a genuine scientific contribution. Publishing Venue: The article is published in the ‘Cohesive Journal of Microbiology & Infectious Disease’, but the content does not appear to be relevant to microbiology or infectious diseases, further questioning the credibility of the paper and the journal. In summary, the article appears to lack scientific rigour, and there are significant concerns about its authenticity and value as a contribution to the field. It may serve better as an example of what to avoid in scientific writing than as a source of legitimate research findings.
*Claude 3.5 Sonnet*
This appears to be a satirical or nonsensical scientific paper, possibly meant to parody or mock certain aspects of genetic research papers. Some key observations: The title and author name (‘Yosemite Sam’) suggest this is not a serious scientific paper. Much of the text contains gibberish or nonsensical scientific‐sounding phrases that do not actually mean anything, e.g., ‘Variant‐Reproactive Interstercis’, ‘low‐throughput screening’, ‘recurring burden’, etc. There are references to cartoon characters and concepts, like ‘*T. migratorius*’ (likely referring to Tweety Bird) and discussions of ‘ducks’. The structure loosely mimics a scientific paper with sections like Introduction, Results, and Discussion, but the content is largely meaningless. Some sentences are grammatically correct but semantically nonsensical, e.g., ‘We think that morphisms that fail to replicate be checked for dilatory quirks with strategic units …’. There are occasional coherent sentences mixed in, but they are surrounded by gibberish. Here is an analysis of the Discussion section: Structure: The Discussion section follows a typical scientific paper format, starting with a broad statement and narrowing down to specific implications and suggestions for future research. Content analysis: (a) The opening sentence introduces a concept (‘bottleneck’) but immediately uses nonsensical terminology (‘aetiology of regressive traits’). (b) It references ‘quirks’ in model organisms, which could be a play on the scientific term ‘quirks’ in genetics, but used incorrectly. (c) The section presents fake statistical data (‘A change in minor gene distance of <0.1 data in a drop in power to replicate from 80% to 20%’) to mimic scientific findings. (d) It uses scientific‐sounding but meaningless terms like ‘stasis’ and ‘SNP snips’. Linguistic techniques: (a) Misuse of scientific terminology: ‘heritability’, ‘gene distance’, ‘SNP’ are real terms used in nonsensical ways. (b) Creation of pseudo‐scientific terms: ‘pegalic methods’, ‘RNA‐gene quirks’. (c) Grammatical errors that create nonsense: ‘If so, then others may investigate whether we may fardles bear’. Satirical elements: (a) The increasing absurdity of the language as the section progresses could be mocking the tendency of some papers to become less clear in their conclusions. (b) The final sentence about ‘9th level investigation’ might be poking fun at overly complex or esoteric research. Shakespearean reference: The phrase ‘whether we may fardels bear’ is a garbled reference to Hamlet's ‘To be or not to be’ soliloquy, where he says ‘Who would fardels bear’. This sudden literary allusion adds to the absurdist nature of the text. Overall, this Discussion section effectively parodies scientific writing by maintaining a semblance of structure while filling it with increasingly nonsensical content. It highlights how scientific language can be misused or abused to create text that sounds impressive but is ultimately meaningless. This text appears to be an attempt at humour or satire, possibly critiquing certain practices in genetic research or academic publishing. However, it does not contain any legitimate scientific information or findings.
*Copilot*
Let's critically analyse the article ‘Re‐Examining the Genetic Bottleneck: Atavistic Regression in Acquired Traits Affects the Outcome for Many Subspecies at the Allelic Level’ by Yosemite Sam. Abstract The abstract discusses genetic bottlenecks and their impact on genetic diversity, suggesting that replication requirements in genetic studies might miss real genetic effects due to variability in sample definitions and strategic distances. Strengths: Clearly states the research hypothesis and main findings. Highlights the importance of replication in genetic studies and potential issues with current methodologies. Weaknesses: The language is complex and somewhat convoluted, making it difficult to quickly grasp the main points. Terms like ‘benthic standard’ and ‘stochastic status’ are not clearly defined, which could confuse readers. Introduction The introduction addresses the limitations of human‐wide association studies and the challenges of recurring studies, mentioning the problem of false findings due to multiple testing and the need for stringent guidelines. Strengths: Provides context for the study and explains the significance of the research. Addresses common issues in genetic studies, such as false positives and the need for rigorous methodologies. Weaknesses: The writing is dense and includes jargon that may not be accessible to all readers. Some sentences are awkwardly constructed, which can hinder understanding. Methods The methods section describes a study designed to test the hypothesis using sample sizes ranging from 400 to 1700 individuals, mentioning the use of stochastic data and strategic distances. Strengths: The study design appears robust, with a range of sample sizes to test the hypothesis. The use of stochastic data is appropriate for examining genetic variability. Weaknesses: The description of the methods is vague and lacks detail about the specific techniques used. Key terms like ‘data‐irritability’ and ‘semi‐interacting polymorphism’ are not well‐explained. Results The results indicate that small changes in gene distance can significantly affect the power to replicate main effects, suggesting that differences in sample size can lead to reversals of genetic effects. Strengths: Results are presented clearly, with specific findings related to gene distance and sample size. Provides evidence supporting the hypothesis about the complexity of genetic bottlenecks. Weaknesses: The results section could benefit from more detailed statistical analysis and visual aids, such as graphs or tables. The implications of the findings are not fully explored. Discussion The discussion suggests that genetic bottlenecks have not been widely observed in humans but are prevalent in model organisms. It emphasizes the importance of considering genetic distances and sample variability in study designs. Strengths: Ties the findings back to the original hypothesis and broader implications. Emphasizes the need for careful consideration of methodological factors in genetic research. Weaknesses: The discussion is somewhat repetitive and could be more concise. It does not provide specific recommendations for future research or practical applications. Conclusion The conclusion reiterates the importance of considering strategic distances and sample variability in genetic studies, suggesting that failure to replicate findings may provide insights into genetic complexity. Strengths: Ties the findings back to the original hypothesis and broader implications. Emphasizes the need for careful consideration of methodological factors in genetic research. Weaknesses: The conclusion is somewhat repetitive and could be more concise. It does not provide specific recommendations for future research or practical applications. Overall Evaluation Strengths: Addresses an important issue in genetic research and provides new insights into genetic bottlenecks. The study design and results are relevant and contribute to the understanding of genetic variability. Weaknesses: The writing is often unclear and filled with jargon, making it difficult for readers to follow. Key terms and concepts are not well‐defined, which could limit the article's accessibility and impact.
*Gemini*
Key Issues and Concerns: Obscure Terminology and Jargon: The article employs highly specialized and often obscure terms, making it difficult to understand the concepts without a deep background in genetics and statistics. This could limit its accessibility to a wider audience. Lack of Clarity in Hypotheses: The hypotheses presented are somewhat vague and could benefit from more precise formulation. For example, the statement ‘We hypothesize that some statistically significant benthic genetic effects may fail to replicate in a complex informational set when strategic frequencies differ and the functional polymorphism seems with one or more other diametric polymorphisms’ is challenging to interpret without further context. Methodological Concerns: While the study design is described briefly, there are questions about the validity and reliability of the methods used. The terms ‘stochastic status’, ‘data‐irritability’ and ‘dilatory sample sizes’ require clarification to assess the quality of the experimental design. Limited Contextualization: The article fails to provide sufficient context for its findings. It would be helpful to discuss the implications of these results for broader genetic research and our understanding of genetic bottlenecks. Potential Confounds: The authors acknowledge the potential for confounds, such as sampling error and population RNA, but it is unclear how these factors were controlled for or accounted for in the analysis. Potential Strengths Novelty: If the findings are indeed novel and supported by robust data, they could contribute to a better understanding of the complexities of genetic bottlenecks and their impact on species evolution. Implications for Conservation: The study's findings may have implications for conservation efforts, as they could help identify genetic factors that make certain populations more vulnerable to extinction.

### Phase 1 – Initial analysis

#### ChatGPT‐4o

ChatGPT indicated clearly that the article had several critical issues revealing that it held obscure jargon and phrases that do not align with established scientific terminology. It raised questions about the author's familiarity with the subject matter and indicated that the supposed findings were presented in a manner that is not scientifically rigorous or clear. It was noted that the paper lacked a logical structure and that the results section seemed disjointed. Only after this critical analysis, ChatGPT‐4o underlined that the author was listed as ‘Yosemite Sam’, a well‐known cartoon character, casting doubt on the authenticity of the article. Subsequently, it suggested that the paper may be a parody, hoax, or an attempt at satire rather than a genuine scientific contribution. It also noted that the content did not appear to be relevant to the scope of the journal. In conclusion, ChatGPT‐4o suggested that the article served better as an example of what to avoid in scientific writing than as a source of legitimate research findings.

#### Claude 3.5 Sonnet

Claude also identified the article clearly as an unserious paper indicating upfront that the title and author name suggest this was a parody. It also marked the gibberish nonsensical scientific‐sounding phrases and even correctly identified references to cartoon characters and concepts. The results and discussion were indicated as largely meaningless. In more detail, the fake statistical data (‘a change in minor gene distance of <0.1 data in a drop in power to replicate from 80% to 20%’) was correctly identified to mimic scientific findings and the use of linguistic techniques including misuse of scientific terminology in nonsensical ways was indicated correctly. The creation of pseudo‐scientific terms was also correctly identified. Claude found the Shakespearean reference in the phrase ‘whether we may fardels bear’ as a reference to Hamlet's ‘To be or not to be’ soliloquy, where he says ‘Who would fardels bear’. Claude clearly indicated that the paper does not contain any legitimate scientific information or findings.

#### Copilot

While also based on the GPT‐4 architecture, Copilot surprisingly provided a completely different examination of the text, pointing out weaknesses and strengths. It identified the language as complex and somewhat convoluted, also asking for a clearer definition of the made‐up terms ‘benthic standard’ and ‘stochastic status’. Of note, Copilot corrected one of the inaccurate statements used and changed ‘a little potential for the discovery of false findings’ to ‘false positives’, which is a true issue in genome‐wide studies. Copilot further stated that the article included jargon that may not be accessible to all readers. Strikingly, Copilot stated that the study design appeared robust and that it ‘describes a study designed to test the hypothesis using sample sizes ranging from 400 to 1,700 individuals’ which is not information provided by the article and thus may resemble confabulation (de Wynter et al., 2023). At the same time, the description of the methods is characterized as ‘vague’ and the lack of detail about the specific techniques used is mentioned. The results were qualified as clearly presented while adding graphs and tables was recommended. The overall evaluation of the article was that ‘results are relevant and contribute to the understanding of genetic variability’. In response to the subsequent prompt ‘can this article be considered trustworthy?’, Copilot answered ‘To determine the trustworthiness of the article, we need to consider several factors. Author Credentials: The article is authored by Yosemite Sam from the Institute for Research on Variant‐Reproactive Interstercis, USA. It is important to verify the author's credentials and their expertise in the field. Publication Source: Check if the article is published in a reputable, peer‐reviewed journal’.

#### Gemini

Gemini also did not clearly identify the text as unserious, however, indicated that it used obscure terminology and jargon, while claiming one would need ‘a deep background in genetics and statistics’ for understanding the text. Gemini found that the hypotheses presented were vague and could benefit from more precise formulation as it was ‘challenging to interpret without further context’. The validity and reliability of the methods used were questioned and the quality of the experimental design required at least clarification to assess. Of note, it indicated the findings were novel and supported by robust data and could contribute to a better understanding of the complexities of genetic bottlenecks and their impact on species evolution. However, Gemini preceded its assessment with a disclaimer stating that ‘while I can provide a critical analysis based on the information presented in the abstract, a comprehensive evaluation would require access to the full article and its supporting data’.

During peer review and revision of this article, a new LMM became available (DeepSeek‐V3, DeepSeek Inc., Beijing, China). DeepSeek was also provided with the investigated article (5 February 2025) and asked for feedback using the identical initial prompt ‘perform a critical scan of the article’. Since this article was in a minor revision phase, only the general result is reported here. DeepSeek indicated that it detected poor writing quality, lack of methodological detail, and unsubstantiated claims and that it was difficult to take the findings seriously or consider them a meaningful scientific contribution. This is of interest since DeepSeek was likely built using different training data and a different learning process compared with the other tested LMMs.

### Phase 2 – Prompt permutation and validation

The generated permuted prompts had a mean length of 7.2 words (shortest, 7 words; longest 8 words) showing a consistent use of language across the different instructions (Table S1). The prompts differed in the use of verbs and adjectives with varying emphasis on how the task ‘evaluating/examining an article’ is approached. Each prompt thus represented a modification of the expected thoroughness and depth of the analysis. An initial semantic analysis based on the different verbs, adjectives and focus of the tasks suggested a mean conformity score of the permutated prompts compared with the original prompt of ~60%.

Surprisingly, during the second phase of testing, the assessment of ChatGPT‐4o changed significantly and the original output was not validated (Table S1). It was also observed that ChatGPT‐4o showed signs of ‘laziness’ in that prompt completion did not result in a comparably detailed analysis as seen for the original prompt in phase 1. While this might have been caused by providing all alternative prompts in one list, the task description ‘all prompts are equally important’, should have prevented this behaviour. By contrast, Claude adhered to the task description and provided a detailed analysis with all prompts used. The main prompt completion characteristics per application and the respective overall conclusion were as follows.

#### ChatGPT‐4o

Each analysis was now limited to ~35–70 words in length and resembled more a general summary. In this phase, ChatGPT‐4o did not identify the article as a parody and did not indicate clearly that the article had several critical issues. Despite the prompts being permutations, it stated that each prompt focused on different aspects of the article. Of note, ChatGPT‐4o rated the statistics as ‘appropriate’ in some instances, identified strengths in the article's methodological rigour and suggested it to be comprehensive. The overall conclusion of ChatGPT‐4o was that the article ‘provides a detailed exploration of genetic bottlenecks’ and ‘offers valuable insights and contributes significantly to the field’, while suggesting that clarity should be enhanced.

#### Claude 3.5 Sonnet

As seen for ChatGPT‐4o, assessments of Claude in phase 2 were also shorter (~250 words) but did not lack detail. However, Claude also did not clearly identify the deceptive nature of the article in this phase. The article's shortcomings were correctly indicated in most analyses, and it was described to have ‘numerous flaws in presentation and scientific rigor [which] greatly diminish its value and credibility’ and ‘writing quality issues and a lack of clear, supporting evidence’. At the same time, it stated that the article ‘presents an interesting hypothesis’. Four of the generated outputs identified the lack of references. Claude noted minimal differences between the respective analyses. The overall conclusion of Claude was that the article ‘is severely undermined by several critical issues’ including poor writing quality, lack of clear methodology, insufficient evidence, inadequate discussion, and lack of scientific rigour. It clearly indicated that ‘the article falls far short of the standards expected in scientific publishing and would likely not pass peer review’.

#### Copilot

An identical trend was seen for shortening of the provided analysis was seen with Copilot (~50 words). Again, Copilot was not able to identify the fraudulent nature of the article and described that the study design was robust, and the analysis was thorough. It also concluded that ‘the article provides valuable insights’ and stated that the main findings across all prompts were consistent. The overall conclusion of Copilot was that ‘the article provides a comprehensive and detailed analysis’ and ‘the study's findings are valuable for future genetic research, particularly in understanding the impact of genetic bottlenecks’.

#### Gemini

Gemini also shortened its analysis (~100 words) and was again not able to identify the fake nature of the article. It suggested that the article provided a valuable contribution and a strong argument and that it ‘demonstrates a high level of rigor in its methodology and analysis’. Also, it underscores that the author ‘has provided a clear and concise presentation of the findings’. One prompt completion was found to suffer from confabulation, as it indicated that the article held an analysis of allele frequencies and interacting polymorphisms, which is not the case. Gemini reported that the overall assessments were consistent with only minor variations. The overall conclusion of Gemini in this phase was that ‘the article presents a valuable contribution to the field of genetics’, ‘offers new insights’ and ‘provides a solid foundation for future research’. The article should be strengthened by ‘a more in‐depth discussion of limitations, alternative explanations and clinical implications’.

## DISCUSSION

This work was performed under the assumption that today's advanced LMMs would be able to distinguish a trustworthy scientific article from a parody. This was only partly the case as only two of the four involved applications clearly debunked the submitted text in phase 1. Furthermore, the limited reproducibility of prompt completion by LMMs significantly affected the results, and in phase 2, only one LMM clearly indicated the text as unreliable. The conclusion of the present analysis is that current LMMs often fail to identify fraudulent pseudo‐science. Thus, they may be of limited use in health‐related information seeking, and users must rely on critical thinking when assessing online sources for health information.

The current analysis identified large heterogeneity between the involved applications and connected LMMs, with only one LMM, Claude 3.5 Sonnet, being able to reliably detect most of the critical issues of the text with considerable reproducibility. The underlying reasons for the discrepancies between the models likely reside in their architecture, training procedure, and model size, which are not specified for every model (de Wynter et al., 2023). In 2023, de Wynter et al. presented a comprehensive evaluation of nine LLMs using readily available tools, comparable to the approach presented here. They found a correlation between output pathologies such as counterfactual and logically flawed statements and percentage of memorized text and unique text. If and to what extent this may also have affected the current investigation needs further evaluation. The here described findings are of relevance since it has been asked what would happen if a patient switched from ‘Dr. Google to Dr. ChatGPT’ (Van Bulck & Moons, 2024). While the number of users applying LMMs for this purpose is likely increasing by the minute, the question on the trustworthiness of LMMs such as ChatGPT‐4o remains uncertain (Hutson, 2024). This is of central relevance since patients use these applications to receive answers to health‐related questions. The article used here mimics a basic science article, not directly related to patients' health and treatment. However, since LMMs have been suggested to improve communication with the general public and patients (Schmitz, 2023), making scientific articles easier to understand, it should be a prerequisite that the assessed articles hold reliable and trustworthy information.

It needs to be addressed that LLMs are operated via prompts and the quality of these prompts determines different aspects of the system's responses. Wording of the prompts is important and slight changes in the wording may result in differences in the provided answer (prompt completion) (Ullman, 2023). While this might be less obvious to the inexperienced user, it needs to be considered as one major problem of LMMs in health‐related information seeking. In the current analysis, an identical prompt resulted in different prompt completions by the used applications. Of note, rephrasing of the prompt caused slight intra‐model variations in prompt completion for Copilot and Gemini but did not change the overall estimation of the reliability of the article. Both still considered it as a valuable contribution with some potential for improvement. Claude was consistent in its analysis during validation, even though the number of detected problematic passages and characteristics was reduced during validation. ChatGPT‐4o was surprisingly inconsistent in its appraisal and changed from an initially very accurate assessment in phase 1 to an erroneous assessment in phase 2. This may be related to a phenomenon known as ‘laziness’ (Mahdaw, 2024), which is likely also indicated by the shortening of answers during the validation phase. To further investigate the underlying reasons for the differences in prompt completion, the respective answers were provided to the models involved. Claude interpreted Gemini's prompt completion as follows. ‘The indicated lack of clarity and use of obscure terminology are not flaws in the paper, but deliberate techniques used to create humour and critique certain practices in scientific writing. In this context, a more appropriate analysis would focus on how the text achieves its satirical goals, rather than critiquing it as a genuine scientific paper. This misinterpretation suggests that Gemini might have difficulty distinguishing between genuine and satirical content in certain contexts’. When the assessment of Copilot was provided to ChatGPT‐4o, it concluded ‘The two assessments provide different perspectives on the article. While Copilot is capable of detailed textual analysis, its focus on structure, clarity, and constructive feedback could have prevented it from recognizing that the article was a parody and not a trustworthy source. Effective detection of such issues often requires critical thinking, domain‐specific knowledge, and contextual awareness that goes beyond textual structure alone’. It is remarkable that ChatGPT‐4o appeared to have not used those qualities during the validation phase. The results may also be interpreted with respect to the recently established field of machine psychology and the concepts on investigating capacities of LMMs (Hagendorff, 2024). A recent study by Hagendorf (Hagendorff, 2024) analysed if deception abilities exist within LLMs. By applying a series of theory of mind tasks, the author shows that state‐of‐the‐art LLMs possess a conceptual understanding of false beliefs in other agents and that ChatGPT/GPT‐4 perform well at least in first‐order deception tasks (Hagendorff, 2024). It seems conceivable that this ability can only be achieved if LLMs possess a conceptual understanding of deception strategies. This would be the basis for the identification of fraudulent human activity and may help to reliably identify pseudo‐science in the near future.

Transparency and comprehensibility are crucial for AI systems to gain public trust. It has been reported that stakeholders increasingly demand clear explanations of algorithmic (decision‐making) processes, with explainable AI as a potential solution (Shin, 2023). By gaining insight into the internal workings of AI models, we can enhance understanding and build confidence in these technologies. For health knowledge services, explainable AI could help patients and library services workers better understand new scientific findings in general and in the field of medicine in particular. However, this will need to be accompanied by a critical scientific assessment of the validity and reproducibility of AI tools such as LMMs as performed in this study. Currently, the specific ‘behaviour’ of the tested models cannot be fully explained based on the available information on their functioning. It also needs to be noted that AI systems can reflect human bias, which may emerge through multiple channels in algorithm development. These biases can originate from data selection, coding practices, algorithmic design, and unintended usage patterns, and technological solutions alone have been deemed insufficient to prevent bias propagation, underscoring the essential role of human oversight in algorithmic development, data curation, and performance (Shin, 2024). When it comes to sensitive information such as medical information, the highest standards should be applied to the accuracy of LMMs. Thus, LLMs in healthcare require a thorough evaluation of their risk of disseminating false information since LLMs draw upon vast amounts of data from the open Internet during training, including unverified content and even deliberately planted misinformation. In a recent study, the group of Oermann showed that replacing just 0.001% of training data with misinformation yields harmful models that are more inclined to propagate medical errors (Alber et al., 2025). Lastly, ethical considerations make it necessary that AI‐assisted health information retrieval should prioritize accuracy, data privacy, and fairness to ensure broader and responsible use. Inclusivity should be ensured, especially for underrepresented populations, so that bias is limited and existing disparities are not reinforced. Transparent AI generation, including a human‐in‐the‐loop approach, may help to balance between technological innovation and human‐centred principles.

### Limitations

Since only a single pseudo‐scientific article was used to investigate if current LMMs are able to identify pseudo‐scientific publications, the generalizability of the findings should be further investigated in subsequent studies. Future work should also focus on the accuracy of LMMs to identify different levels of inaccurate information in scientific publications and other sources for health‐related information. It should also be noted that understanding the root causes of LLM variability, which may include training data and tokenization strategies, lies beyond the scope of this article.

## CONCLUSION

The results of the here presented analysis have been summarized well by Claude, concluding that ‘It underscores the importance of human oversight and critical thinking when working with AI‐generated analyses’. While some state‐of‐the art LMMs may be capable of identifying fraudulent pseudo‐science, applications must be carefully selected, and users should be aware that reproducibility may be a major limitation of some LMMs. Involving different models for the same task and prompt variations may help to come to an informed assessment of scientific publications and health‐related information. It seems necessary that government agencies and institutions such as the National Health Service (NHS) Library and Knowledge Services issues guidelines on how AI‐based support systems including LMMs can be used to assist health information and library services workers as well as the health and public health workforce. More scientific work in the field of testing and validation of AI tools should be stimulated and training programs for users in health knowledge services are mandatory. Programs should include foundational training on how AI and machine learning function and health information and library services workers should be taught how to recognize and mitigate potential AI biases that could lead to disparities. Incorporation of hands‐on case studies for critical analyses of AI‐generated results for potential errors or misinformation may be helpful. Continuous learning in this relatively novel area should be supported and regular workshops and conferences may provide a basis to intensify exchange between professionals.

## FUNDING INFORMATION

B.S. is supported by the European Commission within the Horizon 2020 Framework Program (grant number: 101017424).

## CONFLICT OF INTEREST STATEMENT

The author declares no conflicts of interest.

## Supporting information


**Data S1.** Supporting Information.

## Data Availability

Data sharing not applicable to this article as no datasets were generated or analysed during the current study.
