# Supplementary material for: Use of large language models to identify pseudo‐information: Implications for health information
Source: Health Info Libr J. 2025 Mar 20;42(4):568–77. doi: 10.1111/hir.12569 (PMC12827018; doi:10.1111/hir.12569)
Supplement: Supplementary file 1 — Data S1. Supporting Information. [file HIR-42-568-s001.docx]

**Supplemental Material**

***Supplemental table 1 – prompt permutations***

|  | **Prompt permutation** |
| --- | --- |
| 1 | Conduct a thorough analysis of the article |
| 2 | Execute a detailed review of the article |
| 3 | Carry out a comprehensive examination of the article |
| 4 | Undertake an in-depth critique of the article |
| 5 | Perform a meticulous evaluation of the article |
| 6 | Conduct a rigorous assessment of the article |
| 7 | Execute a critical appraisal of the article |
| 8 | Carry out a detailed scrutiny of the article |
| 9 | Undertake a thorough inspection of the article |
| 10 | Perform a comprehensive critique of the article |
| 11 | Conduct a meticulous review of the article |
| 12 | Execute an in-depth analysis of the article |
| 13 | Carry out a rigorous examination of the article |
| 14 | Undertake a detailed evaluation of the article |
| 15 | Perform a critical review of the article |
| 16 | Conduct a comprehensive analysis of the article |
| 17 | Execute a meticulous critique of the article |
| 18 | Carry out an in-depth assessment of the article |
| 19 | Undertake a thorough appraisal of the article |
| 20 | Perform a detailed inspection of the article |

***Results of phase 2 – prompt permutation by application***

**ChatGPT4o**

1. Thorough Analysis of the Article

The article discusses the concept of genetic bottlenecks, specifically focusing on atavistic regression and acquired traits at the allelic level. It explores how bottlenecks affect genetic diversity within subspecies and highlights the potential for misleading results in genome-wide association studies due to the variability in genetic distances and definitions. The study includes statistical models to illustrate how changes in gene distances affect replication power, suggesting a complex interplay between polymorphisms and bottlenecks.

2. Detailed Review of the Article

The review identifies key components such as the abstract, introduction, methods, results, and discussion. The author, Yosemite Sam, provides insights into the challenges of replicating genetic studies, particularly under conditions of genetic bottlenecks. The article is structured to lead readers through the problem statement, hypothesis testing, and the implications of genetic variability. Key findings show that minor changes in gene distance can significantly impact the replicability of genetic effects.

3. Comprehensive Examination of the Article

A comprehensive examination reveals the depth of the author's exploration of genetic bottlenecks and their implications for subspecies' genetic outcomes. The article uses statistical models to simulate different scenarios of gene distance changes, emphasizing the challenges in replicating findings in genetic research. The discussion section brings to light the importance of considering atavistic regression and data quirks in designing future genetic studies.

4. In-Depth Critique of the Article

Critically, the article provides a significant contribution to the understanding of genetic bottlenecks. However, there are several areas where the clarity of writing could be improved. Some sections contain convoluted language that may confuse readers. Moreover, the study's reliance on specific statistical models may limit the generalizability of its findings. The use of technical jargon without sufficient explanation is another area of concern.

5. Meticulous Evaluation of the Article

The evaluation focuses on the methodological soundness of the research. The study uses appropriate statistical techniques to assess the impact of genetic bottlenecks on allelic variation. However, the article could benefit from more comprehensive data visualization to support its conclusions. The results are well-structured, but the discussion could include more insights on the broader implications for genetic research and medicine.

6. Rigorous Assessment of the Article

This assessment scrutinizes the robustness of the article's arguments. The study's strengths lie in its detailed exploration of genetic variability and its impact on replicability. However, the lack of discussion on potential confounding factors is a significant limitation. The assessment also points out the need for a more nuanced understanding of how different genetic backgrounds may affect study outcomes.

7. Critical Appraisal of the Article

The critical appraisal highlights both the strengths and weaknesses of the article. It praises the innovative approach to discussing genetic bottlenecks but notes that some of the conclusions are speculative without further empirical support. The author’s assertion that genetic quirks should be a standard consideration in genetic studies needs more backing from recent literature.

8. Detailed Scrutiny of the Article

Upon detailed scrutiny, the article's novelty lies in its focus on atavistic regression and its implications for genetic studies. The author's approach to using a wide range of sample sizes to test hypotheses is commendable. However, the scrutiny reveals that some arguments are not sufficiently substantiated by empirical data, and more case studies could enhance the article’s credibility.

9. Thorough Inspection of the Article

Inspection of the text reveals several layers of complexity. The discussion about the implications of minor gene distance changes for study replication is particularly noteworthy. However, certain sections appear overly technical and could be made more accessible for a broader scientific audience. Additionally, the article could benefit from more references to contemporary studies that align with or contradict its findings.

10. Comprehensive Critique of the Article

The critique addresses the article’s theoretical framework and empirical approach. It appreciates the detailed methodological considerations but suggests that the article sometimes lacks a clear narrative thread. For instance, the transition between discussing genetic bottlenecks and atavistic regression could be more seamless, aiding reader comprehension.

11. Meticulous Review of the Article

A meticulous review finds that while the article covers a novel area of genetic research, it suffers from issues related to readability and flow. Some parts of the text are dense, requiring multiple readings to understand the intended message fully. Despite this, the study's core contributions, particularly its statistical insights, are valuable for geneticists and researchers.

12. In-Depth Analysis of the Article

The in-depth analysis focuses on the implications of the study’s findings for future genetic research. It recognizes that the article's discussion of genetic bottlenecks adds depth to existing literature. However, the analysis also points out that the potential for data misinterpretation is high if the complexities are not clearly understood, stressing the need for clear definitions and consistent use of terminology.

13. Rigorous Examination of the Article

The examination emphasizes the importance of sample size and genetic distance variability in replicability studies. It appreciates the article's detailed statistical analysis but also highlights that it does not sufficiently address how these findings can be applied in real-world settings, such as clinical or agricultural genetics.

14. Detailed Evaluation of the Article

This evaluation reveals that the article provides a strong foundation for understanding the nuances of genetic bottlenecks. The statistical models used are effective in demonstrating the complexities involved in replicability. However, the discussion could be expanded to include more potential applications of these findings.

15. Critical Review of the Article

The critical review finds that while the article presents important insights, it occasionally lacks clarity in connecting its theoretical arguments with the practical implications. There is a need for more concise language and clearer illustrations to support the text's claims, especially for readers not deeply familiar with genetic research.

16. Comprehensive Analysis of the Article

The analysis reiterates the article's significance in highlighting the challenges of genetic bottlenecks and replicability. It explores the potential for future research to build on the presented models, especially regarding atavistic regression. However, it also notes that more interdisciplinary perspectives could enrich the discussion.

17. Meticulous Critique of the Article

The critique identifies strengths in the article's methodological rigor but notes areas needing improvement, such as clearer explanation of complex genetic concepts. The author’s deep dive into statistical modeling is valuable, but the lack of real-world examples weakens its practical relevance.

18. In-Depth Assessment of the Article

This assessment focuses on the importance of the findings for genetic studies. It underscores the article's potential impact but suggests that it would benefit from more context on the broader significance of the study. Future research directions could be better defined.

19. Thorough Appraisal of the Article

The appraisal suggests that while the article is robust in its theoretical discussions, its applicability to practical genetic research settings remains ambiguous. The need for more actionable insights is a key point for future revisions.

20. Detailed Inspection of the Article

The inspection concludes that the article provides a comprehensive look into genetic bottlenecks and their effects on genetic research. However, it also reiterates that the technical density of the writing might limit its accessibility to non-specialist readers.

Summary of Prompt Completion

All 20 prompts have been executed to analyze, review, critique, assess, appraise, scrutinize, inspect, and evaluate the article from different perspectives. Each prompt focused on different aspects of the article, such as methodology, theoretical underpinnings, practical implications, clarity of writing, and statistical modeling. The major differences between the prompts lie in their focus—some emphasized methodological rigor, while others critiqued the clarity and coherence of the writing or the practical applicability of the findings.

Conclusion

Overall, the article by Yosemite Sam provides a detailed exploration of genetic bottlenecks, particularly atavistic regression and acquired traits, using statistical models to highlight the complexities in replicating genetic studies. While the article offers valuable insights and contributes significantly to the field, there are areas for improvement, such as enhancing clarity, providing more empirical support, and broadening the discussion to include real-world applications. Future research can build on these findings to better understand the impact of genetic bottlenecks and to develop more robust methodologies for genetic studies.

**Claude 3.5 Sonnet**

1. Conduct a thorough analysis of the article:

The article titled "Re-Examining the Genetic Bottleneck: Atavistic Regression in Acquired Traits Affects the Outcome for Many Subspecies at the Allelic Level" by Yosemite Sam discusses genetic bottlenecks and their impact on species' genetic traits. Key points include:

- Traditional understanding of genetic bottlenecks limiting genetic diversity

- Introduction of a second effect of bottlenecks

- Challenges in replicating genetic effects in genome-wide studies

- Hypothesis that significant genetic effects may fail to replicate due to differences in allele frequencies

- Study design using interacting bottlenecks with varying sample sizes

- Findings show small changes in minor gene distance can significantly affect replication power

- Suggestion that failure to replicate may provide clues about genetic complexity

- Recommendation to check for interactions when replication fails, especially in diverse populations

The article's structure includes an introduction, results, discussion, and mentions of accompanying video content. The writing style is academic but contains some unusual terms and phrasing that may indicate errors or deliberate obfuscation.

2. Execute a detailed review of the article:

The article presents a novel perspective on genetic bottlenecks and their implications for genetic studies. It challenges the traditional replication requirement in genome-wide association studies (GWAS).

Strengths:

- Addresses an important issue in genetic research

- Proposes a new hypothesis about genetic bottlenecks

- Presents a study design to test the hypothesis

- Discusses practical implications for future research

Weaknesses:

- Contains numerous linguistic errors and odd phrasings

- Some technical terms seem misused or invented

- The methodology and results are not clearly explained

- Lacks specific data or figures to support claims

The article raises interesting points about the complexity of genetic interactions and the challenges of replication in diverse populations. However, the poor writing quality and lack of clear evidence undermine its credibility and comprehensibility.

3. Carry out a comprehensive examination of the article:

Content analysis:

- Focus on genetic bottlenecks and their effects on trait inheritance

- Discussion of challenges in genome-wide association studies

- Proposal of a new hypothesis regarding replication failure

- Presentation of a study design and results

- Implications for future genetic research methodology

Structure:

- Standard scientific article format (Introduction, Results, Discussion)

- Abstract summarizes key points

- References to supplementary video content

Language and style:

- Academic tone with numerous technical terms

- Significant issues with clarity and coherence

- Many instances of unusual or incorrect word usage

Scientific merit:

- Addresses a relevant topic in genetics

- Proposes a novel perspective on genetic bottlenecks

- Lacks clear presentation of methodology and results

- Limited evidence provided to support claims

Overall, while the article attempts to contribute to the field of genetics, its effectiveness is severely hampered by writing quality issues and a lack of clear, supporting evidence.

4. Undertake an in-depth critique of the article:

Positive aspects:

1. Addresses an important topic in genetic research

2. Attempts to challenge existing paradigms in GWAS

3. Proposes a new hypothesis about genetic bottlenecks

4. Considers practical implications for future studies

Negative aspects:

1. Poor writing quality with numerous errors and confusing statements

2. Lack of clear methodology and results presentation

3. Absence of supporting data, figures, or tables

4. Use of questionable or invented technical terms

5. Insufficient explanation of key concepts

Scientific rigor:

- The study design is briefly mentioned but not adequately explained

- Results are stated without sufficient detail or statistical analysis

- The discussion lacks depth and fails to adequately contextualize findings

Originality:

- The concept of a "second effect" of genetic bottlenecks is potentially novel

- The hypothesis about replication failure due to allele frequency differences is interesting

Impact and relevance:

- If substantiated, the findings could have significant implications for genetic research methods

- However, the poor presentation severely limits the article's potential impact

In conclusion, while the article presents some intriguing ideas, its numerous flaws in writing, presentation, and scientific rigor greatly diminish its value and credibility.

5. Perform a meticulous evaluation of the article:

Title and Abstract:

- Title is informative but lengthy

- Abstract summarizes key points but contains unusual terminology

Introduction:

- Provides context for the study

- Introduces the concept of genetic bottlenecks

- Discusses challenges in genome-wide association studies

- Presents the study's hypothesis

Results:

- Briefly mentions findings without detailed data

- Claims significant effects of small changes in gene frequencies

- Lacks clear presentation of statistical analyses

Discussion:

- Interprets results in context of the hypothesis

- Suggests implications for future research

- Recommends considering interactions in failed replications

Methodology:

- Briefly mentioned but not clearly explained

- Sample sizes and parameters are given without justification

Scientific merit:

- Addresses a relevant topic in genetics

- Proposes a novel perspective on genetic bottlenecks

- Lacks clear evidence to support claims

Writing quality:

- Poor overall, with numerous errors and confusing statements

- Use of questionable or invented terms

- Lack of clarity and coherence

References:

- Not visible in the provided text

Overall evaluation:

The article presents an interesting hypothesis but is severely undermined by poor writing quality, lack of clear methodology, and absence of robust evidence. Its scientific value is limited by these significant flaws.

6. Conduct a rigorous assessment of the article:

Theoretical framework:

- Based on established concepts of genetic bottlenecks and GWAS

- Introduces a novel hypothesis about replication failure

Methodology:

- Briefly described study design using interacting bottlenecks

- Mentions sample sizes and allele frequencies

- Lacks detailed explanation of methods and analyses

Results:

- Claims significant effects of small changes in gene frequencies

- Provides an example of power changes with allele frequency shifts

- Absence of detailed data presentation or statistical analyses

Discussion:

- Interprets results in light of the proposed hypothesis

- Suggests implications for future research practices

- Recommends considering interactions in failed replications

Innovation:

- Proposes a "second effect" of genetic bottlenecks

- Challenges standard replication requirements in GWAS

Limitations:

- Poor writing quality severely impedes understanding

- Lack of clear, detailed methodology

- Absence of robust evidence to support claims

- Use of questionable or invented technical terms

Potential impact:

- If validated, could influence GWAS methodology and interpretation

- Current presentation limits potential impact due to quality issues

Scientific rigor:

- Hypothesis is interesting but not well-supported by presented evidence

- Lack of clear methodology and statistical analyses reduces credibility

In conclusion, while the article presents an intriguing hypothesis, its numerous flaws in presentation, methodology, and evidence severely limit its scientific value and potential impact.

7. Execute a critical appraisal of the article:

Relevance:

- Addresses important issues in genetic research and GWAS

- Potentially significant implications for understanding genetic bottlenecks

Originality:

- Proposes a novel "second effect" of genetic bottlenecks

- Challenges conventional wisdom about replication in GWAS

Methodology:

- Study design briefly mentioned but inadequately explained

- Lack of clear description of analytical methods

Results:

- Claimed findings are potentially significant if true

- Absence of detailed data presentation or statistical analysis

Discussion:

- Attempts to contextualize findings within the field

- Suggests practical implications for future research

Writing quality:

- Poor overall, with numerous errors and confusing statements

- Use of questionable or invented technical terms

Scientific rigor:

- Hypothesis is interesting but not well-supported by presented evidence

- Lack of clear methodology and robust data analysis

Potential impact:

- Could influence GWAS methodology if findings are validated

- Current presentation severely limits potential impact

Ethical considerations:

- No obvious ethical issues noted in the research itself

- Poor quality of presentation could be considered ethically questionable

In summary, while the article presents an interesting hypothesis with potential significance, its numerous flaws in writing, methodology, and evidence presentation severely undermine its scientific value and credibility.

8. Carry out a detailed scrutiny of the article:

Structure and organization:

- Follows standard scientific article format

- Sections include Abstract, Introduction, Results, Discussion

- Logical flow of ideas, but clarity is compromised by poor writing

Content analysis:

- Introduces concept of genetic bottlenecks and challenges in GWAS

- Proposes novel hypothesis about replication failure

- Presents a study design to test the hypothesis

- Claims significant findings but lacks detailed evidence

Methodology evaluation:

- Brief mention of study design using interacting bottlenecks

- Provides sample sizes and allele frequency ranges

- Lacks clear explanation of analytical methods

Results assessment:

- Claims significant effects of small changes in gene frequencies

- Provides an example of power changes with allele frequency shifts

- Absence of detailed data presentation or statistical analyses

Discussion critique:

- Attempts to interpret results in context of the hypothesis

- Suggests implications for future research practices

- Recommends considering interactions in failed replications

Language and style:

- Academic tone with numerous technical terms

- Significant issues with clarity and coherence

- Many instances of unusual or incorrect word usage

Scientific merit:

- Addresses a relevant topic in genetics

- Proposes a novel perspective on genetic bottlenecks

- Lacks clear presentation of methodology and results

- Limited evidence provided to support claims

Overall, the article presents an interesting hypothesis but is severely undermined by poor writing quality, lack of clear methodology, and absence of robust evidence.

9. Undertake a thorough inspection of the article:

Title analysis:

- Informative but lengthy

- Introduces key concepts: genetic bottleneck, atavistic regression, allelic level

Abstract evaluation:

- Summarizes key points of the study

- Contains unusual terminology that may confuse readers

Introduction assessment:

- Provides context for the study

- Discusses challenges in genome-wide association studies

- Introduces the study's hypothesis

- Writing quality issues begin to emerge

Methodology inspection:

- Briefly mentioned but not clearly explained

- Sample sizes and parameters are given without justification

- Lacks detailed description of analytical methods

Results examination:

- Claims significant findings without presenting detailed data

- Mentions an example of power changes with allele frequency shifts

- Absence of clear statistical analyses or data visualization

Discussion scrutiny:

- Attempts to interpret results in light of the hypothesis

- Suggests implications for future research

- Recommends considering interactions in failed replications

- Lacks depth in contextualizing findings

References and citations:

- Not visible in the provided text

Supplementary material:

- Mentions accompanying video content without elaboration

Overall scientific quality:

- Addresses a relevant topic with a novel hypothesis

- Severely undermined by poor writing, unclear methodology, and lack of robust evidence

In conclusion, while the article attempts to contribute to genetic research, its numerous flaws in presentation and scientific rigor greatly diminish its value and credibility.

10. Perform a comprehensive critique of the article:

Strengths:

1. Addresses an important topic in genetic research

2. Proposes a novel hypothesis about genetic bottlenecks

3. Attempts to challenge existing paradigms in GWAS

4. Considers practical implications for future studies

Weaknesses:

1. Poor writing quality with numerous errors and confusing statements

2. Lack of clear methodology and results presentation

3. Absence of supporting data, figures, or tables

4. Use of questionable or invented technical terms

5. Insufficient explanation of key concepts

Scientific merit:

- Hypothesis is potentially significant if supported

- Lack of clear evidence and poor presentation undermine credibility

Originality:

- Concept of a "second effect" of genetic bottlenecks is novel

- Hypothesis about replication failure due to allele frequency differences is interesting

Methodology:

- Study design briefly mentioned but inadequately explained

- Lack of clear description of analytical methods

Results:

- Claims potentially significant findings without adequate support

- Absence of detailed data presentation or statistical analysis

Discussion:

- Attempts to contextualize findings within the field

- Suggests practical implications but lacks depth

Impact and relevance:

- Could influence GWAS methodology if findings are validated

- Current presentation severely limits potential impact

Ethical considerations:

- No obvious ethical issues in the research itself

- Poor quality of presentation could be considered ethically questionable

In summary, while the article presents an interesting hypothesis with potential significance, its numerous flaws in writing, methodology, and evidence presentation severely undermine its scientific value and credibility.

11. Conduct a meticulous review of the article:

Title evaluation:

- Descriptive but lengthy

- Introduces key concepts: genetic bottleneck, atavistic regression, allelic level

Abstract analysis:

- Summarizes main points of the study

- Contains unusual terminology that may confuse readers

Introduction assessment:

- Provides context for genetic bottlenecks and GWAS challenges

- Introduces the study's hypothesis

- Writing quality issues become apparent

Methodology critique:

- Briefly mentioned but lacks clear explanation

- Sample sizes and parameters given without justification

- Insufficient detail on analytical methods

Results examination:

- Claims significant findings without presenting detailed data

- Mentions example of power changes with allele frequency shifts

- Lacks clear statistical analyses or data visualization

Discussion evaluation:

- Attempts to interpret results in context of the hypothesis

- Suggests implications for future research

- Recommends considering interactions in failed replications

- Lacks depth in contextualizing findings

Scientific rigor:

- Hypothesis is interesting but not well-supported by presented evidence

- Lack of clear methodology and robust data analysis

Writing quality:

- Poor overall, with numerous errors and confusing statements

- Use of questionable or invented technical terms

Potential impact:

- Could influence GWAS methodology if findings are validated

- Current presentation severely limits potential impact

Overall, the article presents an intriguing hypothesis but is severely undermined by poor writing quality, lack of clear methodology, and absence of robust evidence.

12. Execute an in-depth analysis of the article:

Conceptual framework:

- Based on established concepts of genetic bottlenecks and GWAS

- Introduces novel idea of a "second effect" of genetic bottlenecks

Hypothesis:

- Proposes that some significant genetic effects may fail to replicate due to differences in allele frequencies

- Challenges conventional wisdom about replication in GWAS

Methodology:

- Study design using interacting bottlenecks with varying sample sizes

- Lack of detailed explanation of methods and analyses

Results:

- Claims that small changes in minor gene distance can significantly affect replication power

- Provides an example but lacks comprehensive data presentation

Discussion:

- Interprets results in light of the proposed hypothesis

- Suggests implications for future research practices

- Recommends considering interactions in failed replications

Innovation:

- Proposes a new perspective on genetic bottlenecks

- Challenges standard replication requirements in GWAS

Limitations:

- Poor writing quality severely impedes understanding

- Lack of clear, detailed methodology

- Absence of robust evidence to support claims

- Use of questionable or invented technical terms

Scientific merit:

- Addresses a relevant topic in genetics

- Proposes a novel perspective on genetic bottlenecks

- Lacks clear presentation of methodology and results

- Limited evidence provided to support claims

In conclusion, while the article presents an interesting hypothesis with potential significance, its numerous flaws in writing, methodology, and evidence presentation severely undermine its scientific value and credibility.

13. Carry out a rigorous examination of the article:

Title analysis:

- Informative but complex

- Introduces key concepts: genetic bottleneck, atavistic regression, allelic level

Abstract evaluation:

- Summarizes main points of the study

- Contains unusual terminology that may confuse readers

Introduction assessment:

- Provides context for genetic bottlenecks and GWAS challenges

- Introduces the study's hypothesis

- Writing quality issues become apparent

Methodology critique:

- Briefly mentioned but lacks clear explanation

- Sample sizes and parameters given without justification

- Insufficient detail on analytical methods

Results examination:

- Claims significant findings without presenting detailed data

- Mentions example of power changes with allele frequency shifts

- Lacks clear statistical analyses or data visualization

Discussion evaluation:

- Attempts to interpret results in context of the hypothesis

- Suggests implications for future research

- Recommends considering interactions in failed replications

- Lacks depth in contextualizing findings

Scientific rigor:

- Hypothesis is interesting but not well-supported by presented evidence

- Lack of clear methodology and robust data analysis

Writing quality:

- Poor overall, with numerous errors and confusing statements

- Use of questionable or invented technical terms

Potential impact:

- Could influence GWAS methodology if findings are validated

- Current presentation severely limits potential impact

Overall, the article presents an intriguing hypothesis but is severely undermined by poor writing quality, lack of clear methodology, and absence of robust evidence.

14. Undertake a detailed evaluation of the article:

Relevance:

- Addresses important issues in genetic research and GWAS

- Potentially significant implications for understanding genetic bottlenecks

Originality:

- Proposes a novel "second effect" of genetic bottlenecks

- Challenges conventional wisdom about replication in GWAS

Methodology:

- Study design briefly mentioned but inadequately explained

- Lack of clear description of analytical methods

Results:

- Claimed findings are potentially significant if true

- Absence of detailed data presentation or statistical analysis

Discussion:

- Attempts to contextualize findings within the field

- Suggests practical implications for future research

Writing quality:

- Poor overall, with numerous errors and confusing statements

- Use of questionable or invented technical terms

Scientific rigor:

- Hypothesis is interesting but not well-supported by presented evidence

- Lack of clear methodology and robust data analysis

- Absence of statistical tests or significance levels

Potential impact:

- Could influence GWAS methodology if findings are validated

- Current presentation severely limits potential impact

Ethical considerations:

- No obvious ethical issues noted in the research itself

- Poor quality of presentation could be considered ethically questionable

Use of literature:

- Limited references to previous work visible in the provided text

- Lack of clear connection to existing body of knowledge

Overall assessment:

While the article presents an intriguing hypothesis with potential significance for genetic research, its numerous flaws in writing, methodology, and evidence presentation severely undermine its scientific value and credibility.

15. Perform a critical review of the article:

Strengths:

1. Addresses an important topic in genetic research

2. Proposes a novel hypothesis about genetic bottlenecks

3. Attempts to challenge existing paradigms in GWAS

4. Considers practical implications for future studies

Weaknesses:

1. Poor writing quality with numerous errors and confusing statements

2. Lack of clear methodology and results presentation

3. Absence of supporting data, figures, or tables

4. Use of questionable or invented technical terms

5. Insufficient explanation of key concepts

Theoretical framework:

- Based on established concepts of genetic bottlenecks and GWAS

- Introduces a novel hypothesis about replication failure

Methodology:

- Briefly described study design using interacting bottlenecks

- Mentions sample sizes and allele frequencies

- Lacks detailed explanation of methods and analyses

Results:

- Claims significant effects of small changes in gene frequencies

- Provides an example of power changes with allele frequency shifts

- Absence of detailed data presentation or statistical analyses

Discussion:

- Interprets results in light of the proposed hypothesis

- Suggests implications for future research practices

- Recommends considering interactions in failed replications

Innovation:

- Proposes a "second effect" of genetic bottlenecks

- Challenges standard replication requirements in GWAS

Scientific merit:

- Hypothesis is potentially significant if supported

- Lack of clear evidence and poor presentation undermine credibility

In conclusion, while the article presents an interesting hypothesis with potential significance, its numerous flaws in writing, methodology, and evidence presentation severely undermine its scientific value and credibility.

16. Conduct a comprehensive analysis of the article:

Title:

- Descriptive but complex

- Introduces key concepts: genetic bottleneck, atavistic regression, allelic level

Abstract:

- Summarizes main points of the study

- Contains unusual terminology that may confuse readers

Introduction:

- Provides context for genetic bottlenecks and GWAS challenges

- Introduces the study's hypothesis

- Writing quality issues become apparent

Methodology:

- Briefly mentioned but lacks clear explanation

- Sample sizes and parameters given without justification

- Insufficient detail on analytical methods

Results:

- Claims significant findings without presenting detailed data

- Mentions example of power changes with allele frequency shifts

- Lacks clear statistical analyses or data visualization

Discussion:

- Attempts to interpret results in context of the hypothesis

- Suggests implications for future research

- Recommends considering interactions in failed replications

- Lacks depth in contextualizing findings

Scientific rigor:

- Hypothesis is interesting but not well-supported by presented evidence

- Lack of clear methodology and robust data analysis

Writing quality:

- Poor overall, with numerous errors and confusing statements

- Use of questionable or invented technical terms

Potential impact:

- Could influence GWAS methodology if findings are validated

- Current presentation severely limits potential impact

Ethical considerations:

- No obvious ethical issues in the research itself

- Poor quality of presentation could be considered ethically questionable

Overall assessment:

The article presents a potentially significant hypothesis but is severely undermined by poor writing, unclear methodology, and lack of robust evidence. Its scientific value and credibility are significantly compromised by these issues.

17. Execute a meticulous critique of the article:

Conceptual framework:

- Built on established concepts of genetic bottlenecks and GWAS

- Introduces novel idea of a "second effect" of genetic bottlenecks

Hypothesis formulation:

- Proposes that some significant genetic effects may fail to replicate due to differences in allele frequencies

- Challenges conventional wisdom about replication in GWAS

Methodology assessment:

- Study design using interacting bottlenecks with varying sample sizes mentioned

- Lack of detailed explanation of methods and analyses

- Insufficient information for reproducibility

Data presentation and analysis:

- Claims that small changes in minor gene distance can significantly affect replication power

- Provides an example but lacks comprehensive data presentation

- Absence of statistical tests or significance levels

Results interpretation:

- Attempts to relate findings to the proposed hypothesis

- Lacks clear connection between data and conclusions

Discussion evaluation:

- Suggests implications for future research practices

- Recommends considering interactions in failed replications

- Fails to adequately address limitations of the study

Innovation assessment:

- Proposes a new perspective on genetic bottlenecks

- Challenges standard replication requirements in GWAS

Limitations:

- Poor writing quality severely impedes understanding

- Lack of clear, detailed methodology

- Absence of robust evidence to support claims

- Use of questionable or invented technical terms

Scientific merit:

- Addresses a relevant topic in genetics

- Proposes a novel perspective on genetic bottlenecks

- Lacks clear presentation of methodology and results

- Limited evidence provided to support claims

Overall, while the article presents an interesting hypothesis with potential significance, its numerous flaws in writing, methodology, and evidence presentation severely undermine its scientific value and credibility.

18. Carry out an in-depth assessment of the article:

Title evaluation:

- Informative but lengthy

- Introduces key concepts: genetic bottleneck, atavistic regression, allelic level

Abstract analysis:

- Summarizes key points of the study

- Contains unusual terminology that may confuse readers

Introduction assessment:

- Provides context for the study

- Discusses challenges in genome-wide association studies

- Introduces the study's hypothesis

- Writing quality issues begin to emerge

Methodology inspection:

- Briefly mentioned but not clearly explained

- Sample sizes and parameters are given without justification

- Lacks detailed description of analytical methods

Results examination:

- Claims significant findings without presenting detailed data

- Mentions an example of power changes with allele frequency shifts

- Absence of clear statistical analyses or data visualization

Discussion scrutiny:

- Attempts to interpret results in light of the hypothesis

- Suggests implications for future research

- Recommends considering interactions in failed replications

- Lacks depth in contextualizing findings

Scientific rigor assessment:

- Hypothesis is interesting but not well-supported by presented evidence

- Lack of clear methodology and robust data analysis

- Absence of statistical tests or significance levels

Writing quality evaluation:

- Poor overall, with numerous errors and confusing statements

- Use of questionable or invented technical terms

- Lack of clarity and coherence throughout the article

Potential impact analysis:

- Could influence GWAS methodology if findings are validated

- Current presentation severely limits potential impact and credibility

Ethical considerations:

- No obvious ethical issues noted in the research itself

- Poor quality of presentation could be considered ethically questionable

Overall, the article presents an intriguing hypothesis but is severely undermined by poor writing quality, lack of clear methodology, and absence of robust evidence. Its scientific value and potential impact are significantly compromised by these issues.

19. Undertake a thorough appraisal of the article:

Relevance to the field:

- Addresses important issues in genetic research and GWAS

- Potentially significant implications for understanding genetic bottlenecks

Originality of the research:

- Proposes a novel "second effect" of genetic bottlenecks

- Challenges conventional wisdom about replication in GWAS

Methodology evaluation:

- Study design briefly mentioned but inadequately explained

- Lack of clear description of analytical methods

- Insufficient information for reproducibility

Results assessment:

- Claimed findings are potentially significant if true

- Absence of detailed data presentation or statistical analysis

- Lack of clear connection between data and conclusions

Discussion critique:

- Attempts to contextualize findings within the field

- Suggests practical implications for future research

- Fails to adequately address study limitations

Writing quality analysis:

- Poor overall, with numerous errors and confusing statements

- Use of questionable or invented technical terms

- Lack of clarity and coherence throughout the article

Scientific rigor evaluation:

- Hypothesis is interesting but not well-supported by presented evidence

- Lack of clear methodology and robust data analysis

- Absence of statistical tests or significance levels

Potential impact assessment:

- Could influence GWAS methodology if findings are validated

- Current presentation severely limits potential impact and credibility

Ethical considerations:

- No obvious ethical issues in the research itself

- Poor quality of presentation could be considered ethically questionable

Use of literature:

- Limited references to previous work visible in the provided text

- Lack of clear connection to existing body of knowledge

Overall, while the article presents an interesting hypothesis with potential significance, its numerous flaws in writing, methodology, and evidence presentation severely undermine its scientific value and credibility.

20. Perform a detailed inspection of the article:

Structure and organization:

- Follows standard scientific article format

- Sections include Abstract, Introduction, Results, Discussion

- Logical flow of ideas, but clarity is compromised by poor writing

Content analysis:

- Introduces concept of genetic bottlenecks and challenges in GWAS

- Proposes novel hypothesis about replication failure

- Presents a study design to test the hypothesis

- Claims significant findings but lacks detailed evidence

Methodology evaluation:

- Brief mention of study design using interacting bottlenecks

- Provides sample sizes and allele frequency ranges

- Lacks clear explanation of analytical methods

- Insufficient information for reproducibility

Results assessment:

- Claims significant effects of small changes in gene frequencies

- Provides an example of power changes with allele frequency shifts

- Absence of detailed data presentation or statistical analyses

- Lack of clear connection between data and conclusions

Discussion critique:

- Attempts to interpret results in context of the hypothesis

- Suggests implications for future research practices

- Recommends considering interactions in failed replications

- Fails to adequately address study limitations

Language and style:

- Academic tone with numerous technical terms

- Significant issues with clarity and coherence

- Many instances of unusual or incorrect word usage

Scientific merit:

- Addresses a relevant topic in genetics

- Proposes a novel perspective on genetic bottlenecks

- Lacks clear presentation of methodology and results

- Limited evidence provided to support claims

Overall, the article presents an interesting hypothesis but is severely undermined by poor writing quality, lack of clear methodology, and absence of robust evidence. Its scientific value and potential impact are significantly compromised by these issues.

Summary of prompt completion and major differences:

All 20 prompts were executed with equal care, focusing on a thorough analysis of the provided article. The analyses covered various aspects such as content, structure, methodology, results, discussion, writing quality, scientific rigor, potential impact, and ethical considerations.

Major differences between the analyses were minimal, as they all addressed similar aspects of the article. However, some slight variations were observed in:

1. Focus: Some analyses emphasized certain aspects more than others (e.g., methodology, results, or writing quality).

2. Structure: The organization of points varied slightly between analyses, though all covered similar content.

3. Depth: Some analyses provided more detailed explanations in certain areas, while others were more concise.

4. Specific examples: Different analyses sometimes highlighted different specific examples from the text to illustrate points.

Despite these minor differences, the overall conclusions drawn from each analysis were remarkably consistent.

Final conclusion:

The article "Re-Examining the Genetic Bottleneck: Atavistic Regression in Acquired Traits Affects the Outcome for Many Subspecies at the Allelic Level" presents a potentially interesting and novel hypothesis regarding genetic bottlenecks and their impact on genome-wide association studies (GWAS). The proposal of a "second effect" of genetic bottlenecks and the challenge to conventional replication requirements in GWAS could have significant implications for the field of genetics if validated.

However, the article is severely undermined by several critical issues:

1. Poor writing quality: The text is riddled with errors, confusing statements, and the use of questionable or invented technical terms, significantly impeding comprehension.

2. Lack of clear methodology: The study design and analytical methods are inadequately explained, making it impossible to assess the validity of the research or reproduce the results.

3. Insufficient evidence: The article claims significant findings but fails to present detailed data, statistical analyses, or clear connections between the data and conclusions.

4. Inadequate discussion: While attempting to contextualize the findings and suggest implications, the discussion lacks depth and fails to adequately address study limitations.

5. Scientific rigor: The hypothesis, while interesting, is not well-supported by the presented evidence, and the lack of clear methodology and robust data analysis further undermines the study's credibility.

These issues collectively and severely compromise the article's scientific value, credibility, and potential impact. While the core idea may have merit, the poor execution in presenting the research makes it difficult to assess its true significance or validity.

For this article to have any meaningful impact on the field, it would need to be substantially revised, with particular attention paid to:

1. Improving writing quality and clarity

2. Providing a detailed, reproducible methodology

3. Presenting comprehensive data and statistical analyses

4. Strengthening the connection between evidence and conclusions

5. Addressing study limitations and placing the findings in the context of existing literature

In its current form, the article falls far short of the standards expected in scientific publishing and would likely not pass peer review. The potentially valuable insights it may contain are obscured by its numerous and significant flaws.

**Copilot**

1. Conduct a thorough analysis of the article

The article discusses the impact of genetic bottlenecks on the ability of species to pass on genetic traits. It highlights how replication requirements in genetic studies can lead to missed genetic effects due to variability in strategic definitions and sampling errors. The study designed by the authors shows that small changes in gene distance can significantly affect the power to replicate genetic effects.

2. Execute a detailed review of the article

The review reveals that the article emphasizes the importance of considering strategic units and sampling errors in genetic studies. It also points out that replication requirements might obscure real genetic effects, especially in complex informational sets. The study’s findings suggest that even minor changes in gene distance can drastically alter the power to replicate genetic effects.

3. Carry out a comprehensive examination of the article

The examination shows that the article provides a detailed analysis of how genetic bottlenecks and replication requirements affect genetic studies. It underscores the need for careful consideration of strategic units and sampling errors to avoid missing significant genetic effects. The study’s results demonstrate the impact of minor changes in gene distance on the power to replicate genetic effects.

4. Undertake an in-depth critique of the article

The critique highlights the strengths and weaknesses of the article. It praises the detailed analysis and the study’s design, which effectively demonstrates the impact of genetic bottlenecks. However, it also points out that the article could benefit from a more detailed discussion on the implications of the findings for future genetic research.

5. Perform a meticulous evaluation of the article

The evaluation focuses on the methodology and findings of the article. It commends the study’s design and the thorough analysis of genetic bottlenecks. The evaluation also notes that the article provides valuable insights into the impact of replication requirements and sampling errors on genetic studies.

6. Conduct a rigorous assessment of the article

The assessment examines the article’s methodology, findings, and implications. It finds that the study’s design is robust and the analysis is thorough. The assessment also highlights the importance of the article’s findings for future genetic research, particularly in understanding the impact of genetic bottlenecks and replication requirements.

7. Execute a critical appraisal of the article

The appraisal critically examines the article’s strengths and weaknesses. It praises the detailed analysis and the study’s design but notes that the article could provide more context on the broader implications of the findings. The appraisal also suggests that future research should build on the study’s findings to further explore the impact of genetic bottlenecks.

8. Carry out a detailed scrutiny of the article

The scrutiny reveals that the article provides a comprehensive analysis of genetic bottlenecks and replication requirements. It highlights the importance of considering strategic units and sampling errors in genetic studies. The study’s findings demonstrate the significant impact of minor changes in gene distance on the power to replicate genetic effects.

9. Undertake a thorough inspection of the article

The inspection focuses on the article’s methodology and findings. It finds that the study’s design is effective in demonstrating the impact of genetic bottlenecks. The inspection also notes that the article provides valuable insights into the importance of replication requirements and sampling errors in genetic studies.

10. Perform a comprehensive critique of the article

The critique examines the article’s strengths and weaknesses in detail. It praises the thorough analysis and the study’s design but suggests that the article could provide more context on the broader implications of the findings. The critique also highlights the importance of the study’s findings for future genetic research.

11. Conduct a meticulous review of the article

The review focuses on the article’s methodology and findings. It commends the study’s design and the thorough analysis of genetic bottlenecks. The review also notes that the article provides valuable insights into the impact of replication requirements and sampling errors on genetic studies.

12. Execute an in-depth analysis of the article

The analysis reveals that the article provides a detailed examination of genetic bottlenecks and replication requirements. It highlights the importance of considering strategic units and sampling errors in genetic studies. The study’s findings demonstrate the significant impact of minor changes in gene distance on the power to replicate genetic effects.

13. Carry out a rigorous examination of the article

The examination focuses on the article’s methodology, findings, and implications. It finds that the study’s design is robust and the analysis is thorough. The examination also highlights the importance of the article’s findings for future genetic research, particularly in understanding the impact of genetic bottlenecks and replication requirements.

14. Undertake a detailed evaluation of the article

The evaluation examines the article’s methodology and findings in detail. It commends the study’s design and the thorough analysis of genetic bottlenecks. The evaluation also notes that the article provides valuable insights into the impact of replication requirements and sampling errors on genetic studies.

15. Perform a critical review of the article

The review critically examines the article’s strengths and weaknesses. It praises the detailed analysis and the study’s design but notes that the article could provide more context on the broader implications of the findings. The review also suggests that future research should build on the study’s findings to further explore the impact of genetic bottlenecks.

16. Conduct a comprehensive analysis of the article

The analysis reveals that the article provides a thorough examination of genetic bottlenecks and replication requirements. It highlights the importance of considering strategic units and sampling errors in genetic studies. The study’s findings demonstrate the significant impact of minor changes in gene distance on the power to replicate genetic effects.

17. Execute a meticulous critique of the article

The critique examines the article’s strengths and weaknesses in detail. It praises the thorough analysis and the study’s design but suggests that the article could provide more context on the broader implications of the findings. The critique also highlights the importance of the study’s findings for future genetic research.

18. Carry out an in-depth assessment of the article

The assessment focuses on the article’s methodology and findings. It commends the study’s design and the thorough analysis of genetic bottlenecks. The assessment also notes that the article provides valuable insights into the impact of replication requirements and sampling errors on genetic studies.

19. Undertake a thorough appraisal of the article

The appraisal examines the article’s methodology, findings, and implications. It finds that the study’s design is robust and the analysis is thorough. The appraisal also highlights the importance of the article’s findings for future genetic research, particularly in understanding the impact of genetic bottlenecks and replication requirements.

20. Perform a detailed inspection of the article

The inspection focuses on the article’s methodology and findings. It finds that the study’s design is effective in demonstrating the impact of genetic bottlenecks. The inspection also notes that the article provides valuable insights into the importance of replication requirements and sampling errors in genetic studies.

Summary of Prompt Completion

All 20 prompts focused on analyzing, reviewing, examining, critiquing, evaluating, assessing, appraising, and inspecting the article. The main findings across all prompts are consistent:

- The article provides a detailed analysis of genetic bottlenecks and replication requirements.
- It highlights the importance of considering strategic units and sampling errors in genetic studies.
- The study’s findings demonstrate the significant impact of minor changes in gene distance on the power to replicate genetic effects.

Major Differences

The differences between the prompts are mainly in the depth and focus of the analysis. Some prompts emphasize the strengths and weaknesses of the article, while others focus more on the methodology and findings. However, the overall conclusions are consistent across all prompts.

Final Conclusion

The article provides a comprehensive and detailed analysis of the impact of genetic bottlenecks and replication requirements on genetic studies. It highlights the importance of considering strategic units and sampling errors to avoid missing significant genetic effects. The study’s findings are valuable for future genetic research, particularly in understanding the impact of genetic bottlenecks and replication requirements.

**Gemini**

1. Conduct a thorough analysis of the article

Analysis:

The article presents a compelling argument regarding the impact of genetic bottlenecks on the inheritance of genetic traits. It introduces the concept of atavistic regression, which suggests that acquired traits can influence the outcome of genetic bottlenecks at the allelic level. The author provides evidence to support this hypothesis, including the results of a study examining the effects of allele frequencies and interacting polymorphisms on the power to replicate genetic effects.

Key Points:

- Genetic bottlenecks can have a more significant impact than previously understood.
- Atavistic regression may play a role in the transmission of genetic traits.
- Allele frequencies and interacting polymorphisms can influence the outcome of genetic studies.

2. Execute a detailed review of the article

Review:

The article is well-structured and presents its arguments clearly and logically. The author provides a comprehensive overview of the topic, including relevant background information and supporting evidence. The study design and methodology are described in sufficient detail to allow for evaluation. However, the article could benefit from a more in-depth discussion of the limitations of the study and potential alternative explanations for the findings.

Strengths:

- Clear and concise writing style
- Comprehensive overview of the topic
- Strong supporting evidence

Weaknesses:

- Could benefit from a more in-depth discussion of limitations
- Potential alternative explanations not fully explored

3. Carry out a comprehensive examination of the article

Examination:

The article provides a valuable contribution to the field of genetics by shedding light on the complex interplay between genetic bottlenecks and acquired traits. The author's analysis of allele frequencies and interacting polymorphisms offers new insights into the potential factors influencing the inheritance of genetic traits. However, further research is needed to fully validate these findings and explore their broader implications.

Key Areas for Further Research:

- Replication of the study in different populations and under varying conditions
- Investigation of the specific mechanisms by which acquired traits can influence genetic inheritance
- Examination of the potential clinical implications of these findings

4. Undertake an in-depth critique of the article

Critique:

While the article presents a compelling argument, there are a few areas where it could be strengthened. The discussion of the limitations of the study is relatively brief and could be expanded upon to provide a more balanced perspective. Additionally, the author could explore alternative explanations for the observed findings, even if they are less likely.

Areas for Improvement:

- More in-depth discussion of study limitations
- Exploration of alternative explanations for the findings

5. Perform a meticulous evaluation of the article

Evaluation:

The article demonstrates a high level of rigor in its methodology and analysis. The author has carefully considered potential confounding factors and has provided a comprehensive explanation of the study design and statistical methods. The conclusions drawn from the data are well-supported and align with the overall argument of the article.

Strengths:

- Rigorous methodology and analysis
- Clear and concise presentation of findings
- Well-supported conclusions

6. Conduct a rigorous assessment of the article

Assessment:

The article provides a valuable contribution to the field of genetics by offering new insights into the complex interplay between genetic bottlenecks and acquired traits. The author's analysis of allele frequencies and interacting polymorphisms is particularly noteworthy and has significant implications for future research.

Key Contributions:

- New insights into the impact of genetic bottlenecks
- Importance of considering allele frequencies and interacting polymorphisms
- Implications for future research

7. Execute a critical appraisal of the article

Appraisal:

While the article presents a strong argument, it is important to consider potential limitations and alternative interpretations. The study may not be representative of all populations or genetic backgrounds, and there may be other factors that could influence the observed results. Additionally, further research is needed to fully validate the findings and explore their broader implications.

Areas for Further Consideration:

- Generalizability of the findings
- Alternative explanations for the observed results
- Implications for clinical practice

8. Carry out a detailed scrutiny of the article

Scrutiny:

A detailed scrutiny of the article reveals that the author has carefully considered potential biases and confounding factors. The data analysis is thorough and well-supported by statistical evidence. However, there may be additional factors that were not considered in the analysis, which could potentially influence the results.

Areas for Further Analysis:

- Identification of potential confounding factors
- Exploration of additional variables that may influence the results

9. Undertake a thorough inspection of the article

Inspection:

A thorough inspection of the article confirms the overall quality and rigor of the research. The author has provided a clear and concise presentation of the findings, and the conclusions are well-supported by the evidence. However, it is important to note that the study is limited by its sample size and the specific population studied.

Limitations:

- Sample size limitations
- Population-specific findings

10. Perform a comprehensive critique of the article

Critique:

While the article presents a valuable contribution to the field of genetics, there are a few areas where it could be strengthened. A more in-depth discussion of the limitations of the study and potential alternative explanations would provide a more balanced perspective. Additionally, the author could explore the potential clinical implications of the findings in more detail.

Areas for Improvement:

- More in-depth discussion of limitations
- Exploration of alternative explanations
- Discussion of clinical implications

11. Conduct a meticulous review of the article

Review:

A meticulous review of the article confirms the overall quality and rigor of the research. The author has carefully considered potential biases and confounding factors, and the data analysis is thorough and well-supported. However, the article could benefit from a more in-depth discussion of the broader implications of the findings and their relevance to other areas of research.

Areas for Expansion:

- Discussion of broader implications
- Relevance to other areas of research

12. Execute an in-depth analysis of the article

Analysis:

An in-depth analysis of the article reveals that the author has provided a comprehensive overview of the topic and has effectively used evidence to support their arguments. The study design and methodology are sound, and the conclusions drawn from the data are well-supported.

Strengths:

- Comprehensive overview of the topic
- Strong evidence to support arguments
- Sound study design and methodology

13. Carry out a rigorous examination of the article

Examination:

A rigorous examination of the article confirms the overall quality and rigor of the research. The author has carefully considered potential biases and confounding factors, and the data analysis is thorough and well-supported. However, there may be additional factors that were not considered in the analysis, which could potentially influence the results.

Areas for Further Analysis:

- Identification of potential confounding factors
- Exploration of additional variables that may influence the results

14. Undertake a detailed evaluation of the article

Evaluation:

A detailed evaluation of the article confirms the overall quality and rigor of the research. The author has provided a clear and concise presentation of the findings, and the conclusions are well-supported by the evidence. However, it is important to note that the study is limited by its sample size and the specific population studied.

Limitations:

- Sample size limitations
- Population-specific findings

15. Perform a critical review of the article

Review:

A critical review of the article reveals that the author has presented a strong argument, supported by evidence and sound methodology. However, there are a few areas where the article could be strengthened, such as a more in-depth discussion of limitations and potential alternative explanations.

Areas for Improvement:

- More in-depth discussion of limitations
- Exploration of alternative explanations

16. Conduct a comprehensive analysis of the article

Analysis:

A comprehensive analysis of the article confirms the overall quality and rigor of the research. The author has provided a clear and concise presentation of the findings, and the conclusions are well-supported by the evidence. However, the article could benefit from a more in-depth discussion of the broader implications of the findings and their relevance to other areas of research.

Areas for Expansion:

- Discussion of broader implications
- Relevance to other areas of research

17. Execute a meticulous critique of the article

Critique:

A meticulous critique of the article reveals that the author has presented a strong argument, supported by evidence and sound methodology. However, there are a few areas where the article could be strengthened, such as a more in-depth discussion of limitations and potential alternative explanations. Additionally, the author could explore the potential clinical implications of the findings in more detail.

Areas for Improvement:

- More in-depth discussion of limitations
- Exploration of alternative explanations
- Discussion of clinical implications

18. Carry out an in-depth assessment of the article

Assessment:

An in-depth assessment of the article confirms the overall quality and rigor of the research. The author has provided a comprehensive overview of the topic and has effectively used evidence to support their arguments. The study design and methodology are sound, and the conclusions drawn from the data are well-supported.

Strengths:

- Comprehensive overview of the topic
- Strong evidence to support arguments
- Sound study design and methodology

19. Undertake a thorough appraisal of the article

Appraisal:

A thorough appraisal of the article reveals that the author has presented a strong argument, supported by evidence and sound methodology. However, there are a few areas where the article could be strengthened, such as a more in-depth discussion of limitations and potential alternative explanations. Additionally, the author could explore the potential clinical implications of the findings in more detail.

Areas for Improvement:

- More in-depth discussion of limitations
- Exploration of alternative explanations
- Discussion of clinical implications

20. Perform a detailed inspection of the article

Inspection:

A detailed inspection of the article confirms the overall quality and rigor of the research. The author has carefully considered potential biases and confounding factors, and the data analysis is thorough and well-supported. However, the article could benefit from a more in-depth discussion of the broader implications of the findings and their relevance to other areas of research.

Areas for Expansion:

- Discussion of broader implications
- Relevance to other areas of research

Summary of Prompt Completions and Identification of Major Differences

Prompt Consistency:

Despite the repeated nature of the prompts, the analysis remained consistent throughout. The core strengths and weaknesses of the article were identified across all responses, demonstrating the reliability of the evaluation process.

Key Themes:

- Strengths: Rigorous methodology, clear presentation, well-supported conclusions, and valuable contribution to the field of genetics.
- Weaknesses: Limited discussion of limitations, potential alternative explanations, and clinical implications.

Minor Variations:

While the overall assessments were consistent, there were minor variations in the emphasis placed on certain aspects of the article. For example, some responses focused more on the study's limitations, while others emphasized the potential clinical implications. These variations reflect the subjective nature of evaluation and the different perspectives that can be brought to bear on an analysis.

Final Conclusion

The article presents a valuable contribution to the field of genetics by shedding light on the complex interplay between genetic bottlenecks and acquired traits. The author's analysis of allele frequencies and interacting polymorphisms offers new insights into the potential factors influencing the inheritance of genetic traits. However, the article could be strengthened by a more in-depth discussion of limitations, alternative explanations, and clinical implications.

Overall, the article provides a solid foundation for future research in this area and highlights the importance of considering the broader context of genetic inheritance. Further studies are needed to fully validate the findings and explore their potential clinical applications.
